# Genetic Architecture of Familial Hypercholesterolaemia

**DOI:** 10.1007/s11886-017-0848-8

**Published:** 2017-04-13

**Authors:** Mahtab Sharifi, Marta Futema, Devaki Nair, Steve E. Humphries

**Affiliations:** 1 Institute of Cardiovascular Science, University College London, 5 University St, London, WC1E 6JF UK; 2 Department of Clinical Biochemistry, the Royal Free London NHS Foundation Trust, Pond Street, London, NW3 2QG UK

**Keywords:** Familial hypercholesterolaemia, Polygenic hypercholesterolaemia, *LDLR* gene, *APOB* gene, *PCSK9* gene

## Abstract

**Purpose of Review:**

Familial hypercholesterolaemia (FH) is an inherited disorder of low-density lipoprotein cholesterol (LDL-C) which is characterised by a raised cholesterol level from birth and a high risk of premature coronary heart disease. In this paper, we review the genetic basis of FH and its impact on the clinical presentation.

**Recent Findings:**

Mutations in any of three genes (*LDLR*, *APOB* and *PCSK9*) are known to cause autosomal dominant FH, but a mutation can be found in only ∼40% of patients with a clinical diagnosis of FH. In the remainder, a polygenic aetiology is most likely, due to the co-inheritance of common LDL-C-raising variants. The cardiovascular presentation and management of FH will differ between patients based on their underlying genetic factors.

**Summary:**

New genotyping methods such as next-generation sequencing will provide us with better understanding of the genetic architecture of FH.

## Introduction

There are three available criteria for clinical diagnosis of Familial hypercholesterolaemia (FH): the Simon Broome criteria from the UK, the Dutch Lipid Clinic Network criteria from the Netherlands and the MedPed criteria from the USA (Table 1) [2]. In the UK, the National Institute for Health and Care Excellence (NICE) guideline recommends use of the Simon Broome criteria. These criteria include raised cholesterol levels, physical stigmata e.g. tendon xanthomata or an evidence of these signs in first- or second-degree relatives, and having a family history of premature coronary artery disease [3]. A ‘definite’ diagnosis of FH is made if a patient has elevated cholesterol levels and tendon xanthomata or a mutation is found by sequencing a DNA sample from the patient. A ‘possible’ diagnosis of FH is made if the patient has only high levels of cholesterol levels and a family history of hypercholesterolaemia or premature coronary heart disease (CHD). The Dutch Lipid Clinic Network criteria use a point system based on patient’s cholesterol levels, personal and family history of premature coronary artery disease, physical examination and detected mutations to give a possible, probable or definite diagnosis of FH [2]. The Dutch Lipid Clinic Network criteria have been modified by clinicians in Wales to take into account that an elevated triglyceride level in a suspected FH patient makes it less likely that the patient has monogenic FH [1•]. Finally, the MedPed criteria are used for diagnosis of probable FH in the USA and are mainly based on the total cholesterol and low-density lipoprotein cholesterol (LDL-C) cut offs stratified by age and family history. The cut offs are different in individuals with first-, second- and third-degree relatives with FH [4].

**Table 1:** Simon Broome and Dutch Lipid Clinic Network diagnostic criteria for FH diagnostic criteria for index FH individuals

Simon Broome criteria		
Diagnosis of definite FH	Cholesterol concentration (adults >7.5 mmol/l LDL-C^a^ >4.9 mmol/l and children >6.7 mmol/l, LDL-C >4.0 mmol/l)	
	And tendon xanthomata	
	Or DNA-based evidence of a mutation in the *LDLR*, *APOB* or *PCSK9* genes	
Diagnosis of possible FH	Cholesterol concentration (adults >7.5 mmol/l LDL-C^a^ >4.9 mmol/l and children >6.7 mmol/l, LDL-C > 4.0 mmol/l)	
	And at least one of the following below:	
	Family history of myocardial infarction: aged younger than 50 years in second-degree relative or aged younger than 60 years in first-degree relative	
	And/or	
	Family history of raised total cholesterol greater than 7.5 mmol/l in adult first- or second-degree relative or greater than 6.7 mmol.l in child, brother or sister aged younger than 16 years	
Dutch Lipid Clinic Network criteria		Points
Family history	First-degree relative with known premature (men <55 years and women <60 years) coronary and vascular disease or first-degree relative with known LDL-C above the 95th percentile	1
	First-degree relative with tendon xanthomata and/or arcus cornealis, or children aged less than 18 years with LDL-C above the 95th percentile	2
Clinical history	Patient with premature coronary artery disease	2
	Patient with premature cerebral or peripheral vascular disease	1
Physical examination	Tendon xanthomata	6
	Arcus cornealis prior to age 45 years	4
Cholesterol levels	LDL-C ≥ 8.5	8
	LDL-C 6.5–8.4	5
	LDL-C 5.0–6.4	3
	LDL-C 4.0–4.9	1
DNA analysis	Functional mutation in the *LDLR* gene	8
Diagnosis	A ‘definite’ diagnosis requires more than 8 points	
	A ‘probable’ diagnosis requires 6–8 points	
	A ‘possible’ diagnosis requires 3–5 points	
Dutch Lipid Clinic Network criteria modified by clinicians in Wales		Points
	Dutch Lipid Clinic Network criteria plus fasting triglyceride levels	As above
	2.5–3.4 mmol/l	−2
	3.5–4.9 mmol/l	−3
	>5.0 mmol/l	−4
	Genetic testing offered when score—6 points	

^a^If untreated, LDL- C values are unobtainable, see the ‘Correction Factor Table’ in Haralambos et al. [1•] and calculate estimated value

Since FH is a disorder of LDL-C metabolism, it is important to understand the basic process of this pathway. LDL-C particles are comprised of an apoB molecule which envelopes a core of cholesteryl esters and triacylglycerols, together with smaller amounts of other lipid species. During normal lipid regulation, these particles bind to LDL-receptors expressed on the liver surface via their apoB molecule. The binding of LDL-C to its receptor induces a rapid internalisation of the LDL-C particle-receptor complex into the endosome compartment of the cell, where the lipoprotein is broken down into its constituent lipids and amino acids. The LDL-receptor then is either recycled back to the plasma membrane, or diverted to a lysosome and catabolised, so that the LDL-receptor is no longer available for recycling. Defects in any of these processes can therefore potentially cause FH.

## FH-Causing Genes

### The Low-Density Lipoprotein Receptor

The *LDLR* gene was the first gene found where mutations cause FH. It spans 45 kb (kilobases) on the short arm of chromosome 19 and comprises 18 exons that are transcribed and translated into five distinct domains which form the cell surface LDL-receptor [5]. Any defect in the *LDLR* gene can cause loss of function of LDL-receptors resulting in reduced LDL-C uptake from blood and cause FH. In mutation carriers, blood cholesterol level is usually raised two fold above the normal level. In the homozygous form, where two identical mutations have been inherited, one from each parent (usually from a consanguineous marriage), or compound heterozygous FH, where two different mutations on both alleles have been inherited, the cholesterol levels are four or five times greater than those of the heterozygous cases [6].

FH-causing mutations in the *LDLR* gene are found along the entire length of the gene. There are more than 2900 different variants identified in the *LDLR* gene with majority of them being exonic substitutions and small (<100 bp) or large rearrangements (>100 bp) [7••]. More than 90% of the reported variants are likely to be disease causing [8]. Most FH cohort studies showed that among the variants found, a large proportion cluster in exon 4 [9]. This could be due to the large size of exon 4 or to the highly detrimental effect of variants in this exon which encodes the ligand-binding domain, on the gene function compared to variants in other exons. Patients with a mutation in exon 4 might present with more severe FH in the clinics. In contrast, the mutation frequency in exons 15 and 16 is extremely low. The spectrum of FH mutations varies between countries; from Greece, where a relatively small numbers of mutations account for the majority of FH cases, to the Netherlands where the mutation spectrum was found to be extensive [10]. The cause of FH in the UK is highly heterogeneous with over 200 different mutations reported [11, 12] The information regarding molecular diagnosis of FH in some parts of the world such as Latin America and South Asia are scant. In Brazil and Mexico, the countries with the largest cohorts in Latin America, only few *LDLR* mutations have been found that have been encountered in the European population previously [13].

Predicting whether novel variants in *LDLR* are pathogenic or not is not always straightforward, especially for synonymous and missense variants. In 2013, the Association for Clinical Genetic Science (ACGS) published guidelines for the classification of variants, with categories ranging from 1 and 2 (clearly not or unlikely to be pathogenic), to 3 (variants of unknown significance), to 4 and 5 (likely to be or clearly pathogenic). The recently updated *LDLR* variant database with variants classified according to these guidelines may be accessed via: http://databases.lovd.nl/shared/genes/LDLR [7••]. All 128 nonsense substitutions, 336 small frame-shifting rearrangements and 116/117 large rearrangements were considered to be pathogenic (classes 4 and 5). Of the 795 missense variants analysed, 115were in classes 1 and 2, 605 in class 4 and 75 in class 3. One hundred eleven of the 180 intronic variants, 4 of 34 synonymous variants and 14 of 37 reported promoter variants were predicted to be likely or clearly pathogenic (classes 4 and 5). It is clearly of great importance to be able to assess whether variants identified in clinical settings or as incidental findings in genomics projects are pathogenic or not. Although 93% (1588) of *LDLR* variants in the current upgrade of the database have been assigned to an ACGS pathogenicity category, 7% (115) remain as variants of unknown significance. It is hoped that as more information becomes available from in vitro functional studies, the development of additional in silico tools and from the various genomics studies, it will be possible to determine the pathogenicity of these variants, and indeed the classification of some variants may also change as our knowledge increases. The ‘gold standard’ test for pathogenicity of a variant is to carry out co-segregation studies, where the co-inheritance of the variant with elevated LDL-C levels is seen in many relatives in a family, while the relatives without the inherited variant have normal levels of LDL-C. The interpretation of family data may be complicated by the overlay of environmental factors that influence lipid levels and by the presence in the family of other genetic variants that raise or lower LDL-C.

### Apolipoprotein B

Apolipoprotein B (apoB) is the major apolipoprotein on lipoprotein molecules, especially LDL-C, and functions as a ligand to the LDL-receptor. The gene is located on chromosome 2p and spans more than 43 kb. The gene comprises 29 exons and is transcribed and translated into a protein of 4563 amino acids [14]. While truncation mutations in the *APOB* gene cause hypobetalipoproteinemia, mutations causing hypercholesterolaemia are due to missense mutations that result in ligand-defective apoB protein. The LDL-C particles made from this allele are therefore not able to bind to the LDL-receptor and thus accumulate in the blood [15]. A single mutation of the *APOB* gene (p.Arg3527Gln) accounts for approximately 6–10% of all FH cases in European population, and it is located in exon 26 of *APOB* gene [16]. Other *APOB* mutations in other regions of the gene such as p.Arg50Trp, p.Arg1164Thr and p.Gln4494del were also recently found to cause FH [17, 18•]. For other variants, for example for p.Arg3531Cys, which was detected in a patient with a clinical diagnosis of FH, while initial reports showed that LDL-C from the patient had reduced binding to the LDL-receptor, later co-segregation studies found that there was no clear co-segregation [19]. This variant is now considered to be a ‘susceptibility’ variant that raises the likelihood of hypercholesterolaemia in a carrier but does not itself cause frank FH.

### Proprotein Convertase Subtilisin/Kexin Type 9

The *PCSK9* (proprotein convertase subtilisin/kexin type 9) gene encodes an enzyme that is involved in regulating the degradation of the LDL-receptor protein in the lysosome of the cell, preventing it from being recycled to the cell surface. The gene is found on chromosome 1p and comprises 12 exons, covering 39 kb [20]. The PCSK9 molecule is synthesised as an inactive proprotein and undergoes cleavage in the endoplasmic reticulum to produce an enzyme with the prodomain noncovalently bound to the catalytic site, preventing further enzyme action. PCSK9 is secreted mostly from the liver and its binding to the LDL-receptor directs the receptor to the lysosome for degradation [21].

Mutations in the *PCSK9* gene that cause FH are gain-of-function mutations that increase LDL-receptor degradation and consequently reduce the number of receptors on the cell surface. Although more than 20 such variants have been reported world-wide, the only common *PCSK9* variant in the UK is p.Asp374Tyr, which occurs in about 2% of the mutation-positive FH patients. This variant is associated with a raised cholesterol level and a high risk of developing premature coronary heart disease, compared with a mutation in the *LDLR* gene [22]. On the other hand, loss-of-function mutations that inactivate the PCSK9 protein lead to less degradation of the LDL-receptor [23]. The most common of these variants, p.Arg46Leu, enhances the clearance of LDL-C from the plasma and lowers cholesterol level in the plasma. In European populations, approximately 3% of individuals are carriers of this variant, and because of their lifelong lower LDL-C levels, they have ∼28% lower CHD risk [24].

### Other Monogenic Causes of FH

A very rare autosomal recessive hypercholesterolaemia is caused by mutations in the low-density lipoprotein receptor adaptor protein 1 (*LDLRAP1*) gene which encodes a cytosolic protein that interacts with the cytoplasmic tail of the LDL-receptor. Mutations in this gene that usually cause premature truncations of the protein lead to LDL-receptor malfunction and hypercholesterolaemia. This gene is located on the short arm of chromosome 1 [25]. The LDL-C level in these cases is typically intermediate between homozygote and heterozygote autosomal dominant FH patients [2].

Several studies have reported that a specific mutation (p.Leu167del) in *APOE* gene causes autosomal dominant FH [26]. This mutation has been previously reported to be associated with sea-blue histiocytosis and familial combined hyperlipidaemia (FCH) but overlap between the FCH and FH phenotype has been shown before as hypertriglyceridemia can be seen due to many common genetic and environmental factors [27, 28].

Several studies have been conducted to identify new genes causing FH, using family studies and next-generation sequencing (NGS) and this has identified three genes *STAP1* (signal transducing adaptor protein family 1), *LIPA* (lysosomal acid lipase) and *PNPLA5* (patatin-like phospholipase-domain-containing family) where mutations may be causing significantly elevated LDL-C and possibly the clinical phenotype of FH [29•, 30, 31]. So far, for *STAP1* and *PNPLA5*, these genes and variants in them have yet to be independently confirmed as FH-causing.

## Frequency of FH

Next-generation sequencing has driven major advances in our understanding of monogenic cause of elevated LDL-C and premature CHD, focussing on the three proven FH-causing genes (*LDLR*, *APOB* and *PCSK9*). Although the prevalence of FH has historically been estimated at 1/500, the likely true prevalence of FH-causing mutation carriage now appears to be between 1/250 and 1/300 in many European populations. In Denmark, 98,098 participants from the Copenhagen General Population Study were genotyped for the three common *LDLR* mutations and commonest *APOB* mutation (p.Arg3527Gln). The prevalence of the four FH mutations was 1/565, accounting for ∼39% of pathogenic mutations in the country, and equating to a total prevalence of FH-mutation carriers of 1/217 [32••]. A similar prevalence was reported in subjects in the UK 10,000 genome project [33], and in a large sample of healthy subjects in the USA [34]. In support of this higher estimate, although historically the prevalence of homozygote FH is believed to be 1 per million [6], several population-based studies have now estimated the prevalence to be around 1/300 with a mean LDL-C levels prior to lipid-lowering treatment being 12.9 ± 5.1 mmol/l. [35] This higher figure would be expected if the true prevalence of FH mutation carriers is 1/250.

The frequency of heterozygous FH is also considerably higher in some populations due to founder effect. This occurs when immigration of a small number of subjects to a geographical area is followed by a population expansion from those individuals. If, by any chance, those individuals have FH, then genetic drift could lead to a high proportion of affected people in that population. Such founder effects have been reported in French Canadian, South African-Afrikaners, Jews and Indians and Finns [2].

## Polygenic versus Monogenic FH

**Fig. 1:**
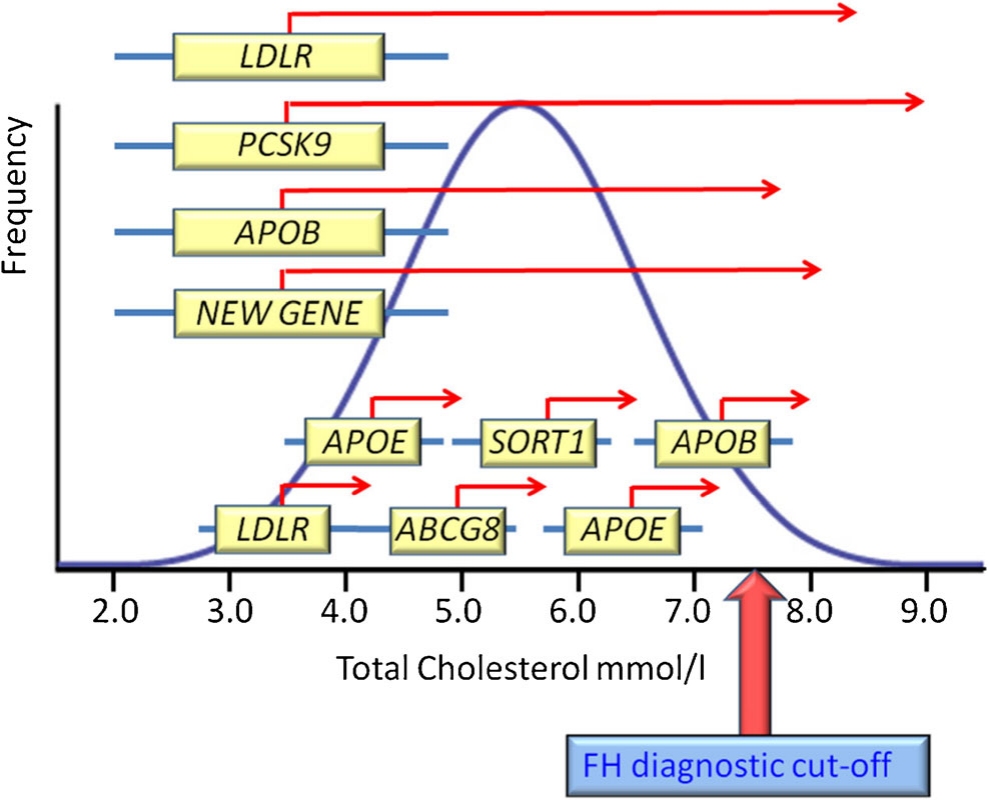
Monogenic and polygenic causes of elevated plasma total cholesterol. Mutations that cause loss of function in LDLR or APOB or gain of function in PCSK9 result in an individual moving from a low point in the population cholesterol distribution to being over the diagnostic cut-off for familial hypercholesterolaemia (7.5 mmol/l). Mutations in PCSK9 are the most severe and in APOB, the mildest. The possibility that mutations in *STAP1* may cause FH is shown. These individuals have ‘monogenic FH.’ It is also possible to have total cholesterol levels above the FH diagnostic cut-off by having inherited a greater than average number of common cholesterol-raising variants (SNP2, SNP2 etc) each of modest effect. As shown in (*5*), key SNPs are in LDLR, APOB, APOE (2×) ABCG8 and SORT1. These individuals have a ‘polygenic’ cause of their hypercholesterolaemia. In more than 80% of those with a clinical diagnosis of FH but with no detectable mutation in LDLR/APOB/PCSK9, the polygenic explanation is most likely. In the remainder, mutation in a novel gene may be present

In diagnostic laboratories, a mutation in one of the three known FH-causing genes can be found in 60–80% of patients with a clinical diagnosis of definite FH and 30% of patients with possible FH [11]. As shown in Fig. 1, mutations that cause loss of function in *LDLR* or *APOB* or gain of function in *PCSK9* result in an individual moving from a low point in the population cholesterol distribution to being over the diagnostic cut-off for FH (7.5 mmol/l). In those where a causative mutation cannot be found, there is a strong possibility that there may be a polygenic cause for FH. The Global Lipids Genetics Consortium meta-analysis identified over 100 loci where common variants influence LDL-C levels [36]. Thus, in patients where no mutation can be found, the LDL-C and total cholesterol level is raised above the FH diagnostic cut-off by having inherited a greater than average number of common cholesterol-raising variants with modest effect. Such key single nucleotide polymorphisms (SNPs) are located in *LDLR*, *APOB*, *APOE*, *ABCG8* and *SORT1*. In more than 80% of those with a clinical diagnosis of FH but with no detectable mutation in *LDLR/APOB/PCSK9*, the polygenic explanation is most likely. In the remainder, mutation in a novel gene may be present [37••, 38•].

The additional polygenic contribution might explain the variation in the LDL-C concentrations among the family members of the FH patients. In these families, it might be possible that the FH phenotype is caused by the combination of a single mutation of large effect in *LDLR/APOB/PCSK9* and several LDL-C alleles of modest effect which may differ in the family members [37••].

## Cascade Testing

All recent European guidelines for the management of FH patients have recommended that healthcare professionals should offer a referral for cascade testing to every FH patient to identify affected first- and second- degree relatives of the index patient [3, 39]. DNA testing would thus confirm the diagnosis of FH and helps to identify affected relatives at an early age. Lipid-lowering therapy in these individuals results in reducing the morbidity and mortality from premature CHD and would be a highly cost-effective strategy in the health services [40, 41]. Such cascade testing of the relatives of affected individuals has been carried out effectively in several countries in Europe, including Holland, and shown to be feasible in the UK [40].

The ascertainment of index cases in the UK is currently relying on a case presenting with premature CHD or the incidental finding of an extreme total cholesterol (TC) or LDL-C level. One problem is that TC and LDL-C values in adults with FH overlap substantially with values observed among individuals from the general population, where a higher than average burden of common, small-effect cholesterol-raising alleles can mimic the biochemical features of FH [37••]. Thus, biochemical screening for FH in adulthood is prone to misclassification. For this reason, according to recent surveys and a national audit, the FH patients currently treated by lipid clinics in the UK represent <15% of an estimated 126,000 FH patients in the UK (if the incidence of 1/500) or <7.5% of the estimated 252,000 FH patients (if the true incidence is 1/250).

## Reverse Cascade Testing

Wald and colleagues used the diagnostic criteria of either total cholesterol >5.31 mmol/l (95th percentile) plus one mutation, or two cholesterol values of ≥5.90 mmol/l (99th percentile), in 10,094 children in the UK at the time of routine immunisations (median age 12.7 months) to identify adults with FH by measuring cholesterol level [42••]. They identified 45 children with FH, including 37 with a detected mutation and 8 with a raised LDL-C but with no mutation. The prevalence of mutation carriers was estimated at 1/273 (37/10,940). We have previously demonstrated that such individuals (without a monogenic cause of FH) are likely to have a polygenic aetiology for their FH phenotype, with co-inheritance of a greater than average number of common LDL-C-raising variants of modest effect, and have proposed that only those patients with a detected mutation should be designated as monogenic FH. Testing parents of the confirmed FH children identified 40 parents who also met the criteria for FH diagnoses.

In the UK, in FH patients where a monogenic cause is found, roughly 93% have a mutation in the *LDLR* gene, 5% in *APOB* and 2% in *PCSK9* [43]. In the Wald study, 40% of the detected FH-mutations were the *APOB* (p.Arg3527Gln) mutation which is known to cause a generally less severe FH than most *LDLR* mutations. Thus, while this screening approach has value in finding new families with FH, it will also identify individuals with less severe form of hypercholesterolaemia and at lower cardiovascular risk. In these patients, the genetic data may contribute to decision-making for intensive statin treatment, but the risk-benefit balance is not as clear as for relatives of clinically diagnosed FH index cases, whose personal or family history of premature CHD is a key diagnostic component.

## Cardiovascular Disease in FH Patients with Different Genetic Cause of their Disease

The earlier studies of heterozygous FH patients, before statin therapy became a standard treatment, showed the risk of fatal or nonfatal coronary heart disease by the age of 60 years was about 50% for male and 30% for females compared with 10% in the relatives without FH [2]. The concept of a cumulative LDL-C burden since birth may play an important role in the aetiology of cardiovascular disease in monogenic FH patients. Traditional cardiovascular risk factors such as age, male gender, smoking and hypertension may play additional roles to the genetic defects in increased coronary risk in these patients [44, 45].

Severity of the clinical presentations is different among the FH patients [39]. The severity and clinical expression of CHD are even variable within a family, where all relatives carry the same *LDLR* gene defect [46–48]. The *LDLR* mutations (null allele) that severely impair the function of LDL-receptor are shown to cause more advanced CHD with earlier onset [46, 48, 49]. In addition, the severity of atherosclerosis appears to be greater in monogenic FH than that of polygenic hypercholesterolaemia [50•, 51]. Aortic valve and supravalve calcification is common among the FH patients with *LDLR*-negative mutations and homozygous FH [52, 53].

This elevated risk for CHD in FH patients with a detected mutation has been convincingly confirmed by Khera et al. in a population-based analysis [54••]. Using NGS for the known FH genes among 20,485 CHD-free individuals, 1386 (6.7%) had LDL-C >4.9 mmol/l, and of these, 24 (1.7%) carried a known FH mutation. Compared with individuals with LDL-C <3.7 mmol/l and no mutation, those with LDL-C >4.9 mmol/l and no FH mutation had a six-fold higher risk for CHD, but those with both LDL-C >4.9 mmol/l and an FH mutation had a 22-fold higher risk. This risk is explained by the substantially higher accumulated ‘LDL-C burden’ since patients have had genetically determined lifelong high LDL-C.

## Conclusion

Monogenic FH is mainly caused by mutations in common FH-causing genes. In patients with a clinical diagnosis of FH where no mutation found, it is most likely to be a polygenic cause for the clinical presentation. Using new genotyping methods such as NGS produces a large amount of sequence data which must be analysed using statistical and bioinformatics approaches and has increased the number of occasions where a variant of uncertain significance is identified. This creates a diagnostic conundrum which requires either in vitro molecular analysis to examine the impact on transcription or splicing, or co-segregation study of families to see if other relatives with the same variant have also high LDL-C levels. Better screening programmes for diagnosis of FH are needed in the community and for cascade testing of the relatives of the FH patients.
